# DENSE: efficient and prior knowledge-driven discovery of phenotype-associated protein functional modules

**DOI:** 10.1186/1752-0509-5-172

**Published:** 2011-10-24

**Authors:** Willam Hendrix, Andrea M Rocha, Kanchana Padmanabhan, Alok Choudhary, Kathleen Scott, James R Mihelcic, Nagiza F Samatova

**Affiliations:** 1 Department of Computer Science, North Carolina State University, Raleigh, 27695, USA; 2 Computer Science and Mathematics Division, Oak Ridge National Laboratory, Oak Ridge, 37831, USA; 3 Department of Civil and Environmental Engineering, University of South Florida, Tampa, 33620, USA; 4 Department of Electrical Engineering and Computer Science, Northwestern University, Evanston, 60208, USA; 5 Department of Integrative Biology, University of South Florida, Tampa, 33620, USA

## Abstract

**Background:**

Identifying cellular subsystems that are involved in the expression of a target phenotype has been a very active research area for the past several years. In this paper, *cellular subsystem *refers to a group of genes (or proteins) that interact and carry out a common function in the cell. Most studies identify genes associated with a phenotype on the basis of some statistical bias, others have extended these statistical methods to analyze functional modules and biological pathways for phenotype-relatedness. However, a biologist might often have a specific question in mind while performing such analysis and most of the resulting subsystems obtained by the existing methods might be largely irrelevant to the question in hand. Arguably, it would be valuable to incorporate biologist's knowledge about the phenotype into the algorithm. This way, it is anticipated that the resulting subsytems would not only be related to the target phenotype but also contain information that the biologist is likely to be interested in.

**Results:**

In this paper we introduce a fast and theoretically guranteed method called *DENSE *(Dense and ENriched Subgraph Enumeration) that can take in as input a biologist's *prior *knowledge as a set of query proteins and identify all the dense functional modules in a biological network that contain some part of the query vertices. The density (in terms of the number of network egdes) and the enrichment (the number of query proteins in the resulting functional module) can be manipulated via two parameters γ and *μ*, respectively.

**Conclusion:**

This algorithm has been applied to the protein functional association network of *Clostridium acetobutylicum *ATCC 824, a hydrogen producing, acid-tolerant organism. The algorithm was able to verify relationships known to exist in literature and also some previously unknown relationships including those with regulatory and signaling functions. Additionally, we were also able to hypothesize that some uncharacterized proteins are likely associated with the target phenotype. The DENSE code can be downloaded from http://www.freescience.org/cs/DENSE/

## 1 Background

Application of genomic and systems-biology studies towards environmental engineering (e.g., waste treatment) generally requires understanding of microbial response and metabolic capabilities at the genome and metabolic levels. This includes understanding of relationships between phenotypes and the various cellular subsystems. In biological systems, phenotype-related genes encode for a number of functionally associated proteins that may be found across a number of different metabolic, regulatory, and signaling pathways [1,2]. Together these pathways form a biologically important network of proteins (or genes) that are responsible for the expression of a particular phenotype. Through analysis of biologically conserved network models, insights into the functional role of phenotype-related genes and functional associations between these genes in these networks can be obtained. This knowledge can then be used by metabolic engineers to identify which genes are potential candidates for modification studies and to determine how modification of selected genes could impact the desired outcome (e.g., hydrogen production). Proteins encoded by these phenotype-related genes can be present in a number of biochemical reactions, pathways, or motifs; understanding of the role and interactions of these proteins within various networks is necessary to identify which cellular subsystems are important for enhancing or suppressing expression of phenotypic traits. Typically, clustering can be used to partition an organism's biological network into interacting protein subgraphs that can further be analyzed for phenotype-relatedness. However, traditional, "hard" clustering results in a partitioning of the data into non-overlapping clusters. And since proteins may belong to multiple cellular subsystems, an approach that allows for overlapping clusters is more appropriate than the one that partitions the data. Retrieving all overlapping clusters from the data not only increases the complexity of the problem, but most of the resulting clusters maybe irrelevant to the phenotype's expression. The complexity and the quality of the results can be improved if a biologist's "*prior *knowledge" about the phenotype can be directly incorporated into the search. For example, a biologist might wish to search an organismal protein functional association network for those modules associated with motility using some of the known flagella proteins as "*prior *knowledge" or a biologists may use the enzymes in the TCA cycle pathway to identify subsystems related to aerobic respiration. Those proteins with unknown functions in the resulting subnetworks would likely have a function related to motility (or aerobic respiration) and may be appropriate for experiments and further inquiry. In this paper, we describe a theoretically sound and fast method called the Dense ENriched Subgraph Enumeration (DENSE) algorithm that capitalizes on the availability of any "prior knowledge" about the proteins involved in a particular process and identifies overlapping sets of functionally associated proteins from an organismal network that are enriched with the given knowledge. When applied to a network of functionally associated proteins in the dark fermentative, hydrogen producing and acid-tolerant bacterium, *Clostridium acetobutylicum*, the algorithm is able to predict known and novel relationships, including those that contain regulatory, signaling, and uncharacterized proteins.

## Results and Discussion

### Description of the Clostridium acetobutylicum ATCC 824 network

The gene functional association network for *Clostridium acetobutylicum *ATCC 824 was obtained from the STRING database [3]. The nodes in the networks are genes that encode enzymes, regulatory proteins, signaling proteins, and others. An edge is placed between a pair of genes if there is some evidence that they are functionally associated. STRING builds these networks based on various lines of evidence, including gene fusion, co-occurrence across species, and co-expression under similar experimental conditions.

### Biological Relevance

To discover clusters related to phenotypes and sub-phenotypes associated with hydrogen production from waste materials, the DENSE algorithm was applied to the hydrogen producing bacterium, *Clostridium acetobutylicum *ATCC 824. *C. acetobutylicum *is a widely studied and well-characterized organism for hydrogen production in nutrient-rich systems [4,5]. In addition to dark fermentative hydrogen production, *C. acetobutylicum *exhibits a number of phenotypes important for bacterial growth and for production of hydrogen. Such phenotypes include dark fermentative hydrogen production and acid-tolerance down to pH of 4.4-6.0 [6]. While *Clostridium *species are often associated with dark fermentative acidogenesis, they are also known for production of solvents [6,7]. During solventogenesis, hydrogen produced is consumed and butanol, ethanol, and acetone are generated [6]. The following sections present a description of biological networks identified and predicted interactions between proteins (and genes) that play a role in uptake and production of hydrogen through regulation, signaling, or synthesis of key enzymes. Specifically, emphasis is placed on key proteins and networks identified in the previous methodologies (e.g, hydrogenases or enzymes for butyrate production). To identify dense, enriched protein-protein interaction networks, three experiments were conducted. In the first experiment, proteins directly related to the [FeFe]-hydrogenase (HydA) were identified. In the last two experiments, hydrogen-related and acid-tolerant knowledge priors identified using the statistical Student's t-Test and our method for discovery of phenotype-related metabolic pathways [8] method were incorporated into the algorithm and clusters were analyzed.

### Dark fermentative hydrogen production

In fermentative hydrogen-producing organisms, such as *C. acetobutylicum*, hydrogen yields are dependent on the presence and activation of hydrogen producing enzymes, called hydrogenases [9]. Studies evaluating the role of hydrogenase in hydrogen production have shown that organisms can contain more than one type of hydrogenases that can each require sets of accessory proteins for activation. As such, the presence or absence of specific accessory proteins plays an important role in regulating the activity of hydrogenase and hydrogen production or uptake in microorganisms. In addition, many hydrogenases are thought to either directly or indirectly regulate other metabolic processes, such as nitrogen metabolism [10]. Therefore, understanding of phenotype-related proteins required for activation and maturation of hydrogenases is important for metabolic engineering of organisms.

#### Hydrogenase

**Figure 1:**
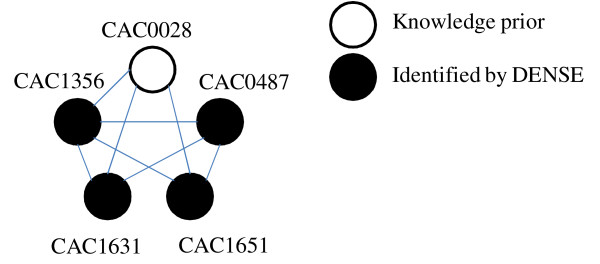
**DENSE cluster containing hydrogenase and associated proteins identified by DENSE**.

**Figure 2:**
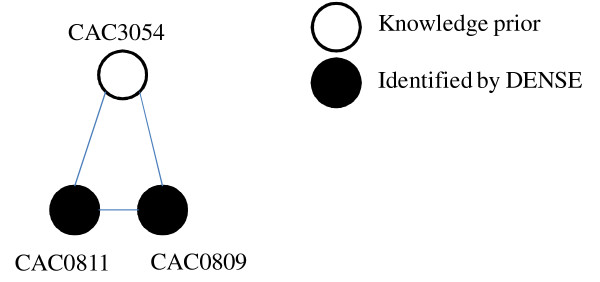
**DENSE cluster containing phosophoheptose and interacting proteins identified by DENSE algorithm**.

When applied to HydA, a hydrogen producing hydrogenase enzyme, the DENSE algorithm was able to identify three maturation proteins that are essential for expression of a [FeFe]- hydrogenase [11]. They are HydE (CAC1631), HydF (CAC1651), and HydG (CAC1356) (Figure 1; Table 1). When these proteins are present and interact with HydA1, activation of the hydrogen producing [FeFe]-hydrogenase occurs. According to studies on hydrogenases, deletion of one of the proteins will result in inactivation of the [FeFe]-hydrogenase [11]. In addition to identifying key protein clusters, the algorithm predicted an association between an uncharacterized protein (Figure 1; CAC0487) and the three maturation proteins. According to the STRING database, CAC0487 is an uncharacterized protein. Since CAC0487 is highly interconnected with the maturation proteins, it can be predicted that the protein is involved in development of the [FeFe]-hydrogenase (HydA1). Utilizing this information, the role of CAC0487 in relation to the three maturation proteins could be characterized through genetic studies and then applied to bioengineering hydrogen producers. Application of the algorithm using hydrogen-related enzymes identified with Schmidt *et al *[8] resulted in prediction of over 6,000 clusters (see Additional File 1) of phenotype-related protein-protein functional associations. Of these clusters, a number of protein functional association networks containing proteins associated with expression of key enzymes related to either hydrogen uptake were identified. Examples of enzymes include those involved in maturation of hydrogenase (HypE and HypD) and nitrogenase (Nif), and key fermentation pathways for hydrogen production in anaerobic organisms. Within these clusters, both known and new associations between proteins involved in regulation, synthesis, and signalling of hydrogen producing pathways are identified. Review of our predicted protein-protein association clusters for the hydrogen production phenotype revealed the presence of only one cluster containing known hydrogenase proteins (Figure 2; Table 2). Within this cluster are two [NiFe]-maturation hydrogenase proteins (HypE and HypD) and phosphoheptose isomerase (GmhA). HypD (CAC0811) and HypE (CAC0809) proteins are depicted as associated, further strengthening the importance of [NiFe]-maturation proteins in impacting the overall hydrogen yields in hydrogen-producing organisms. Since Hyp proteins are involved in activation and synthesis of uptake hydrogenase enzymes [9], down-regulation of HypD and HypE in *Clostridium *species are potential targets for enhancing biological hydrogen production. The HypABC proteins, HypD and HypE are together functionally important for expression of the [NiFe]-hydrogenase and deletion of one of the proteins may lead to inactivation [9]. While the interaction between the two Hyp proteins is clearly defined by previous studies [9,12,13], their interaction with phosphoheptose isomerase is not well understood. Phosphoheptose isomerase or GmhA (CAC3054) is an enzyme involved in biosynthesis of glycerol-manno-heptose [14]. In *Escherichia coli*, phosphoheptose isomerase is involved in biosynthesis of ADP-L-glycero-*β*-D manno-heptose, a compound required in development of lipopolysaccharide (LPS) [14,15]. Specifically, ADP-L-glycero-*β*-D manno-heptose utilized in biosynthetic pathways resulting in production of S-layer glycoproteins and production of the inner-core of LPS [15]. While development of lipolysaccharides is typically found in gram negative bacteria, the presence of LPS in *Clostridium *has been reported [15]. According to the results, all three proteins are shown to be functionally associated with one another (Figure 1). However, from Figure 2, it is unclear why and how the two hydrogenase proteins (HypD and HypE) interact with GmhA.

**Table 1:** Protein-protein functional association network corresponding to Figure 1 and description of hydrogenase-related proteins present in *Clostiridum acetotbutylicum*

STRING ID	Protein ID	Protein Description
CAC0028	HydA1	Hydrogenase I (Hydrogene dehydrogenase)

CAC0487	-	Uncharacterized protein

CAC1651	HydF	Predicted GTPase with uncharacterized domain

CAC1631	HydE	Biotin synthase family enzyme

CAC1356	HydG	Thiamine biosynthesis enzyme

**Table 2:** Protein-protein functional association network corresponding to Figure 2 and description of hydrogenase-related proteins present in *Clostiridum acetotbutylicum*

STRING ID	Protein ID	Protein Description
CAC3054	GmhA	Phosphoheptose isomerase

CAC0811	HypD	Hydrogenase expression-formation factor

CAC0809	HypE	Hydrogenase formation factor

#### Pyruvate: Ferredoxin Oxidoreductase and Associated Proteins

**Figure 3:**
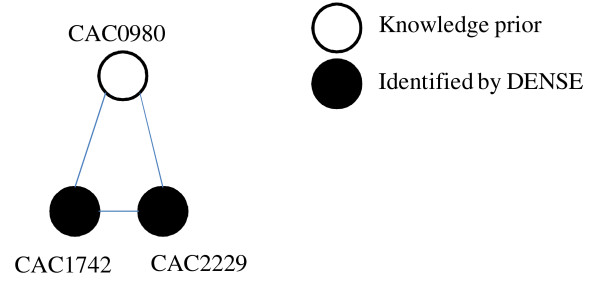
**DENSE cluster containing pyruvate-ferredoxin oxidoreductase and interacting proteins identified by DENSE algorithm**.

Another important enzyme for hydrogen production in *C. acetobutylicum *is pyruvate: ferredoxin oxidoreductase (CAC2229). In anaerobic, hydrogen-producing organisms, pyruvate: ferredoxin oxidoreductase or PFOR is responsible for the conversion of pyruvate to acetyl-CoA [16-18]. Acetyl-CoA is then utilized by a number of pathways, including acetate and butyrate fermentation routes. During production of acetate and butyrate, hydrogen is also produced as a by-product. In this regard, the DENSE algorithm was able to predict the association of this important enzyme when pyruvate lyase was given as a hydrogen-related knowledge *prior *enzyme. While pyruvate formate lyase (PFL) is utilized to generate formate and acetyl coenzyme A (Acetyl-CoA) in facultative anaerobic bacteria [16], it is not uncommon to find genes encoding PFL in anaerobic organisms, such as *Clostridium *[19]. In this study, many clusters containing PFL were identified, but only one that contained PFOR. Figure 3 andTable 3 demonstrate an example of one cluster containing PFL (CAC0980) identified by the DENSE algorithm. In this cluster, the algorithm identified interactions between the two acetyl-CoA forming enzymes, PFL and PFOR (CAC2229) and a third enzyme involved in the acetyl-CoA pathway--phosphotransacetylase (CAC1742). Phosphotransacetylase (Pta) is involved in the conversion of acetyl-CoA to acetyl-phosphate [20]. Interactions between phosphotransacetylase and PFOR are consistent with known biochemical data. Although the presence of PFOR and PFL has been described in *Clostridium*, the direct interaction between the two enzymes is not well known. In *C. acetobutylicum*, PFOR is involved in the pathway for acetyl-CoA and acetogenesis [20]. However, PFL, if utilized, may be involved in production of other products, such as solvents, through alternative pathways.

**Table 3:** Pyruvate: Ferredoxin oxidoreductase and associated proteins present in *Clostiridum acetobutylicum*

STRING ID	Protein ID	Protein Description
CAC0980	-	Pyruvate-formate lyase

CAC2229	-	Pyruvate:ferredoxin oxidoreductase

CAC1742	Pta	Phosphotransacetylase

#### Butyrate Kinase and Associated Proteins

During dark fermentative hydrogen reactions, such as those that occur in anaerobic wastewater reactors, acetic acid and butyric acid are the two metabolites, sought after by scientists and engineers. One reason for this is that through production of these two metabolites hydrogen gas is also co-evolved as a by-product. Therefore, through production or absence of acetate or butyrate by microorganisms, scientists could verify if metabolic fluxes are directed towards hydrogen production rather than hydrogen consumption. As such, understanding the mechanisms involved in production of acetic acid (acetate) or butyric acid (butyrate) is important for enhancing hydrogen production yields.

**Figure 4:**
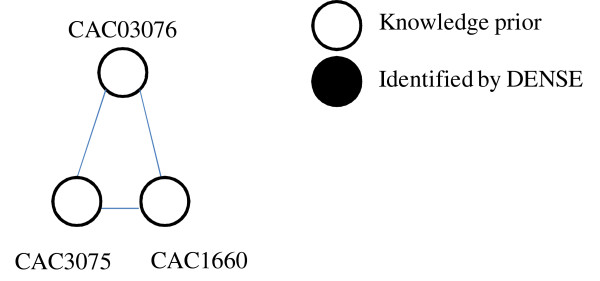
**DENSE cluster containing butyrate kinase enzymes and phosphate butyryltransferase identified by DENSE**.

In this study, application of the DENSE algorithm resulted in identification of a number of clusters including proteins involved in acetate and butyrate formation. From the results, one cluster that contained butyrate kinase, a key enzyme in butyrate formation was identified. Within this cluster, two butyrate kinase proteins (CAC1660 and CAC3075) and one phosphate butyryltransferase (CAC3076) protein are predicted as associated with one another (Figure 4; Table 4). Such associations between these two proteins are consistent with known biochemical data regarding butyrate formation [20]. In these studies, both butyrate kinase and phosphate butyryltransferase (Ptb) are described as essential for production of butyric acid [21]. While associations between the proteins do not appear to be trivial, it is important to note the involvement of Ptb in regulation of metabolic shifts between butyrate and butanol formation. In *C. acetobutylicum*, the switch between acidogenesis and solventogenesis has been shown to occur after formation of butyanol-CoA. In studies evaluating activities of the two enzymes, potentially important feedback mechanisms between the activity of Ptb and butyrate formation, and between Ptb and ATP formation were detected [21,22]. One example of a feedback mechanism is the inhibition of Ptb by ATP during butyrate formation [21]. Based on these flux studies, researchers suggested that Ptb may serve a regulatory role as a signaling protein. When additional interactions between Ptb and other proteins are evaluated, results predicted that Ptb also interacts with two aldehyde dehydrogenases (AdhE2) and acetyl-CoA dehydrogenase. During solvent production, AdhE proteins are responsible for butanol production. Since *C. acetobutylicum *is capable of both solventogenesis and acidogenesis, and Ptb is interacting with proteins involved in both butyrate and butanol formation, it can be hypothesized that Ptb is responsible for metabolic shifts involving butyrate fermentation.

**Table 4:** Description of butyrate kinase and associated proteins present in *Clostiridum acetobutylicum*

STRING ID	Protein ID	Protein Description
CAC3076	Ptb	Phosphate butyryltransferase

CAC1660	Buk	Butyrate kinase, buk

CAC3075	Buk	Butyrate kinase, BUK

### Acid-Tolerance

**Figure 5:**
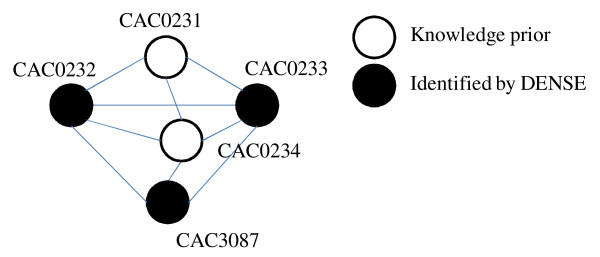
**DENSE cluster containing phosphotransferase system (PTS) enzymes identified by DENSE algorithm**.

Incorporation of acid-tolerant knowledge priors identified by the Student's t-Test and Schmidt *et al *[8] for the dark fermentative, acid-tolerant, hydrogen producing bacterium, *Clostridium acetobutylicum *resulted in identification of 889 dense, enriched protein-protein clusters (see Additional File 2). Due to limitations in identifying a diverse set of completely sequenced organisms, the acid-tolerant proteins incorporated are representative of a small subset of acid-tolerant organisms from the *Phylum Firmicutes *(9 species) and *Proteobacteria *(1 species). As such, the clusters identified are based on organisms representative of three classes of bacteria--Bacilli, Clostridia, and *α*-proteobacteria. Of these clusters, the DENSE algorithm identified 158 as containing proteins involved in a sugar phosphotransferase system (PTS). PTS is a system consisting of a number of proteins involved in uptake of sugar (e.g., glucose and fructose) [23]. Each of these proteins are divided into one of two components--E1 and E2. The E1 component consists of two proteins, E1 enzyme and histidine (Hpr), is responsible for phosphorylation of substrates within the system [23,24]. The E2 component contains the cytoplasmic proteins, EIIA, EIIB, and EIIC. In Figure 5 andTable 5 a densely enriched cluster of PTS proteins identified by DENSE is presented. Proteins involved in this cluster include E1 proteins (CAC0231), EII enzymes (CAC0233 and CAC0234), a transcriptional regulator involved in sugar metabolism (CAC0231), and fructose 1-phosphate kinase (CAC0232). The EII proteins and fructose 1-phosphate kinase are shown to interact with each protein in the cluster. Whereas the transcriptional regulator and EI protein are the only two proteins that are not directly associated. This suggests that the transcriptional regulator is likely involved in controlling the interactions between the cytoplasmic proteins in PTS and fructose 1-phosphate kinase. Fructose 1-phosphate kinase is responsible for conversion of D fructose 1-phsophate to fructose 1,6 biphosphate [23]. Thus, the regulator may play a role in regulating sugar metabolism in *C. acetobutylicum*. While PTS and sugar metabolism are thought of as involved in acid tolerance, literature reports for acid response mechanisms in *Escherichia coli *and *Streptococcus sobrinus *suggested that proteins associated with PTS were upregulated during growth at low pH (pH *<*6.0) [24,25]. In a study by Nasciemento *et al*. [24], PTS activity was shown to be upregulated in *S. sobrinus *when cells were exposed to a pH of 5.0. However, they found the opposite to be true for *Streptococcus mutans*, with PTS activity decreasing by half when exposed to a pH of 5.0. For *E. coli*, Blankenhorn *et al*. [25] showed the phosphocarrier protein PtsH and the protein N(pi) phosphohistidine--sugar phosphotransferase (ManX) were induced by *E.coli *during acid stress. While there is no consistent reaction to acid stress by organisms regarding sugar metabolism and PTS, it does appear that PTS in *C. acetobutylicum *is regulated by a transcriptional factor. Since hydrogen production studies often rely on utilization of glucose (and fructose) as their carbon source, understanding the metabolic response to acid is important. As such, studies evaluating the role of the transcription regulator (CAC0231) on PTS and sugar metabolism in *C. acetobutylicum *under varying pH conditions are necessary.

**Table 5:** Description of acid tolerent cluster identified by DENSE

STRING ID	Protein ID	Protein Description
CAC0233	-	PTS system, IIA component

CAC0231	-	Transcriptional regulator of sugar metabolism

CAC3087	-	Phosphoenolpyruvate-protein kinase (PTS system enzyme I)

CAC0232	-	1-phosphofructokinase (fructose 1-phosphate kinase)

CAC0234	-	PTS system fructose-specific IIBC component

### Effectiveness of DENSE at Efficiently Detecting *μ*, *γ*-quasi-cliques

**Figure 6:**
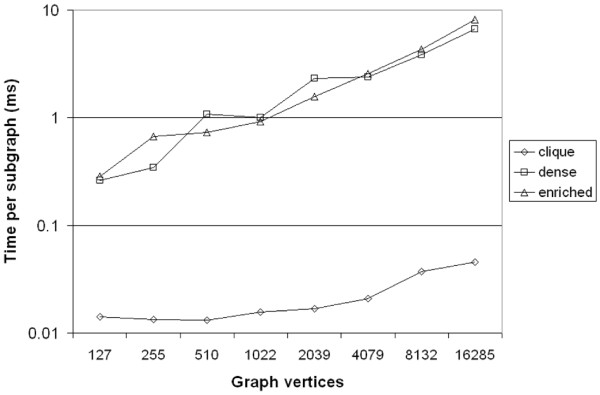
**Timing results for *μ*, *γ*-quasi-clique enumeration algorithm**. Time is reported in milliseconds per quasi-clique. Descriptions of the various quasi-cliques can be found in Table 6, and descriptions of the graphs used can be found in Table 7.

**Figure 7:**
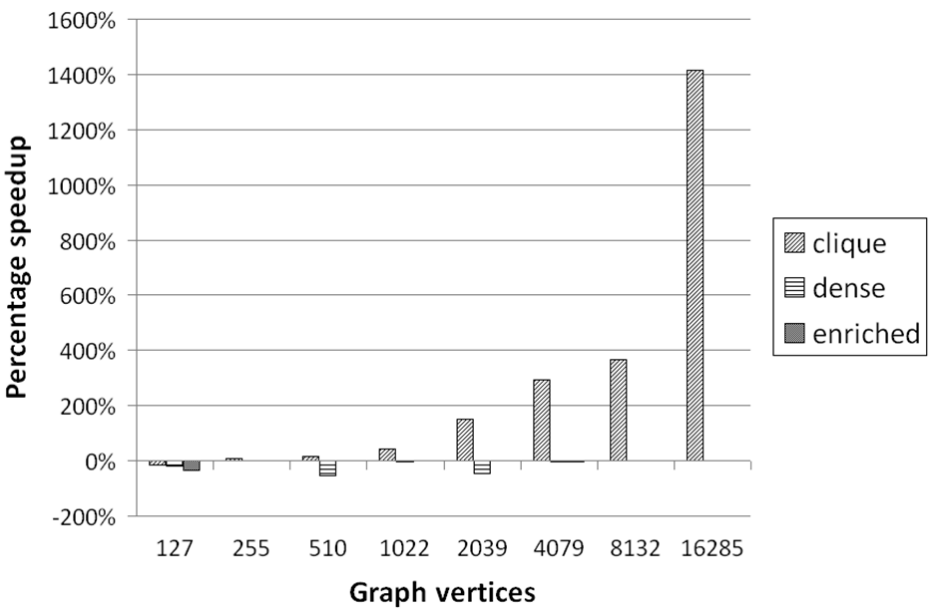
**Speedup results for using hierarchical bitmap index in *μ*, *γ*-quasi-clique enumeration algorithm**. Speedup is reported in percentage; i.e., a value of 100% indicates that using the hierarchical bitmap index was twice as fast as the implementation with the flat index, and a value of -100% indicates that using the flat bitmap index was twice as fast as the implementation with the hierarchical index.

In this section, we present several empirical results to demonstrate the effectiveness of our algorithm at efficiently detecting dense and enriched subgraphs in large, sparse graphs. For these experiments, we ran our algorithm three times in order to detect different types of *μ*, *γ*-quasi-cliques. The three types of quasi-cliques we detect are: high density, low enrichment ("clique") subgraphs where *Q *contains every vertex of the graph; high enrichment, low density ("enriched") subgraphs with a small query set (every 10th vertex of *V *(*G*)); and moderate enrichment and density ("dense") subgraphs with a medium-sized query set (every 6th vertex of *V *(*G*)). These settings were chosen to test the algorithm (and various candidate vertex constraints) under a wide variety of conditions. The parameter settings for these three types of subgraphs appear in Table 6. For these experiments, we used the R-MAT random graph generator [26] to generate sparse graphs of increasing size. The graphs were generated to have vertices equal to a power of two, with an average vertex degree of 14 (|*E*(*G*)| = 7|*V *(*G*)|). The graphs were then processed to remove isolated vertices, which do not contribute to our search for dense, enriched subgraphs. All graphs were generated using the default R-MAT parameters of *a *= 0.45, *b *= 0.15, *c *= 0.15, and *d *= 0.25. More details on the generated graphs can be found in Table 7. For our implementation, we select the candidate vertex to add to the subgraph using a trivial heuristic: the candidate that appears first in the array is chosen. We tested our algorithm on the R-MAT graphs described in Table 6 using all three of the parameter settings in Table 7 and we calculated the rate at which the *μ*, *γ*-quasi-cliques were produced. The results appear in Figure 6. From Figure 6, we can see that the "clique" subgraphs were generated much more quickly than the "dense" or "enriched" quasi-cliques, likely due to the extremity of the density requirement for the "clique" subgraphs, which ensures that the resulting quasi-cliques are fully connected. Also notable is that the time required per quasi-clique appears to increase linearly on the log plot, implying that the time per quasi-clique increases polynomially with the size of the graph. Using a best fit curve, we see that the time per "clique" quasi-clique increases at a rate of approximately *O*(*n*^0.25^), where *n *is the number of vertices in the graph, and the time per "dense" and "enriched" quasi-clique increases at a rate of approximately *O*(*n*^0.65^). Thus, we can estimate the time complexity as approximately *O*(*kn*^0.25^) for the "clique" subgraphs and *O*(*kn*^0.65^) for the "dense" and "enriched" subgraphs, where *k *is the number of subgraphs produced. While this scaling is obviously dependent on the graphs being analyzed, this result does suggest that our algorithm would be able to efficiently calculate dense and enriched subgraphs on large, sparse graphs with a power-law degree distribution. As a second experiment, we wished to evaluate the effectiveness of using the hierarchical bitmap index described in the methods section. For the purposes of this test, we implemented a second version of the algorithm that used only a flat (non-hierarchical) bitmap index, and we compared the time per quasi-clique for both implementations. The results appear in Figure 7.

**Table 6:** Parameter settings for the various types of dense, enriched subgraphs to test DENSE

Description	*γ*	*μ*	|*Q*|
clique	0.999	0.001	|*V*(*G*)|

enriched	0.5	0.90	|*V*(*G*)|/10

dense	0.85	0.85	|*V*(*G*)|/6

**Table 7:** Graph size and number of maximal quasi-cliques for graphs generated using R-MAT

Graph size		Quasi-cliques		
**|*V*(*G*)|**	**|*E*(*G*)|**	**clique**	**enriched**	**Dense**

127	889	569	23	14

255	1785	1199	64	21

510	3570	2593	104	72

1022	7154	5563	270	257

2039	14273	11831	485	432

4079	28553	24930	943	659

8132	56924	52025	1915	1774

16285	113995	106973	3991	4031

32526	227682	219092	8158	8307

From Figure 7, we can see that as the size of the graph increases, the hierarchical bitmap index provides a significant speedup in the rate of identifying "clique" subgraphs. When calculating "dense" and "enriched" subgraphs, the flat index offers a moderate improvement over the hierarchical index (as much as 53%), though this advantage disappears on graphs larger than 2,048 vertices. These results are likely due to the fact that the graphs in question have significantly more "clique" subgraphs than "dense" or "enriched" subgraphs--as the size of the index grows, so does the potential advantage in using a hierarchical index. As such, we conclude that the hierarchical index is successful at improving the algorithmic runtime as the size of the index grows.

## Conclusion

In this paper we describe an algorithm to identify subgraphs from organismal networks with density greater than a given threshold and enriched with proteins from a given query set. The algorithm is fast and is based on several theoretical results. We show the application of our algorithm to identify phenotype-related functional modules. We have performed experiments for two phenotypes (the dark fermenation, hydrogen production and acid-tolerence) and have shown via literature search that the identified modules are phenotype-related.

## Methods

**Figure 8:**
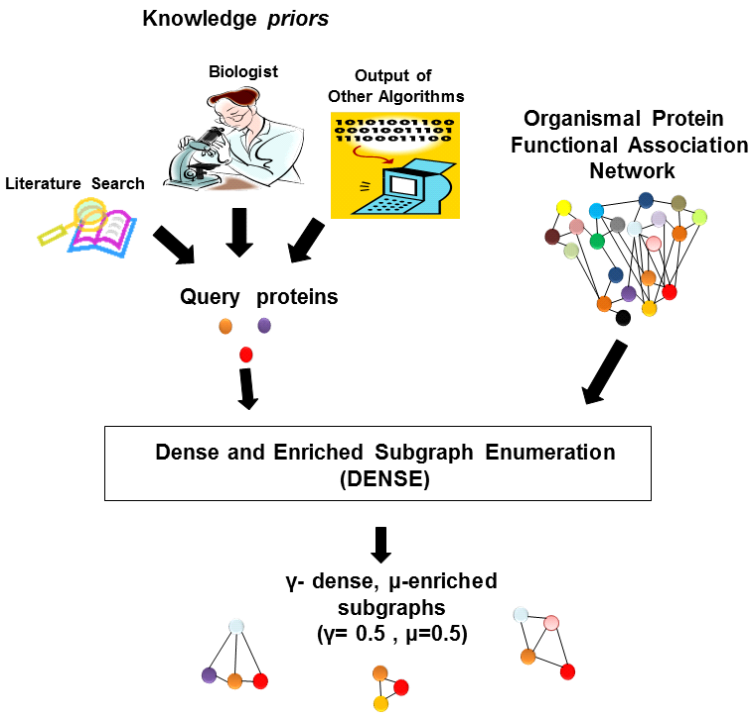
**Overview of the DENSE algorithm**.

Given a phenotype-expressing organism, the DENSE algorithm (Figure 8) tackles the problem of identifying genes that are functionally associated to a set of known phenotype-related proteins by enumerating the "dense and enriched" subgraphs in genome-scale networks of functionally associated or interacting proteins. A "dense" subgraph is defined as one in which every vertex is adjacent to at least some *γ *percentage of the other vertices in the subgraph for some value *γ *above 50%, which corresponds to a set of genes with many strong pairwise protein functional associations. The researchers' prior knowledge is incorporated by introducing the concept of an "enriched" dense subgraph in which at least *μ *percentage of the vertices are contained in the knowledge prior query set. Genes contained in such dense and enriched subgraphs, or *μ*-enriched, *γ*-dense quasi-cliques, have strong functional relationships with the previously identified genes, and so are likely to perform a related task. Previous approaches to finding such clusters have included fuzzy logic-based approaches [27] (also, see [28]), probabilistic approaches [29,30], stochastic approaches [31], and consensus clustering [32]. The discovery of dense non-clique subgraphs has recently been explored by a number of other researchers [33-38], and a number of different formulations for what it means for a subgraph to be "dense" have emerged.

Luo *et al *[39] discuss 3 types of dense subgraphs other than cliques: *k*-plexes, *k*-cores, and *n*-cliques. The *k*-plexes [40] are subgraphs where each vertex is connected to all but *k *others. More specifically, Luo *et al *[39] use a *k*-plex definition where *k *= *n*/2. A definition similar to *k*-plex has been used by Carter and Johnson [35]. Meanwhile, *k*-cores [41] are subgraphs where each vertex is connected to at least *k *others, and *n*-cliques [42] are subgraphs with diameter at most *n*. In this paper we use a more restrictive definition of the *n*-clique, i.e, 2-clique with some additional constraints. Abello *et al *[33] use a definition where at least $\gamma \left(\begin{array}{c} n\\ 2 \end{array}\right)$ edges exist in the subgraph, and Bu *et al *[34] use a definition of a dense subgraph based on the eigenvalue decomposition of the adjacency matrix of the graph. Gao and Wong [36] use a definition based on "clique percolation," meaning that any dense subgraph must satisfy the property that one could reach all of the vertices by taking a clique of size 4 in the subgraph and changing one vertex at a time to form another clique of size 4 until every vertex has been touched. Pei *et al *[37] and Zeng *et al *[38] describe cross-graph quasi-cliques, which use a similar notion of subgraph density as we do, but their work describes techniques for finding subgraphs that meet this density criterion across several graphs at once, whereas we are interested in quasi-cliques that are "enriched" with respect to some knowledge priors. In this paper, we attempt to outline theoretical conditions on dense subgraphs of a network that are enriched with respect to some target set of vertices. An algorithm based on this theory would be able to answer "fuzzy queries" on graph data, identifying dense, possibly overlapping subgraphs in which the "query set" of vertices is overrepresented. By finding these dense, enriched "fuzzy clusters," or enriched quasi-cliques, we hope to achieve superior precision and coverage over conventional hard clustering techniques, which heuristically partition graphs into non-overlapping subgraphs. Further, by limiting the focus to discovering those "quasi-cliques" in which the query labels are overrepresented, the search space for identifying these quasi-cliques may be limited, which has the potential to improve execution time significantly over full quasi-clique enumeration. In this work, we use the following definition for a "dense" subgraph:

**Definition 1.1 ***Given a labeled graph G and a real value γ *??#8712; (0.5, 1], *a subgraph S of G is a **γ-dense quasi-clique **if and only if every vertex of S is adjacent to at least γ*(|*S*| - 1) *of the other vertices of S. If γ*(|*S*| - 1) *is not a natural number, every vertex would need to be adjacent to *??#8968;*γ*(|*S*| -1)??#8969; *of the other vertices of S*.

There are two advantages of using this definition. First, it corresponds nicely with the typical use of the term "density" in that it forces a certain fraction of the possible edges in the subgraph to exist. The second advantage is that by framing the definition as a condition that each vertex must satisfy, we force the resulting subgraphs to be "uniformly" dense. As an illustration, a graph consisting of an isolated vertex and a subgraph in which every pair of vertices is connected may contain a high overall percentage of the possible edges, but it is unlikely anyone would consider the isolated vertex to be related to the others in any significant sense.

**Definition 1.2 ***Given a labeled graph G, a "query" set of vertices Q, a real value γ *??#8712; (0.5, 1], *and a real value μ *??#8712; (0, 1], *a γ-dense quasi-clique S is **μ-enriched **with respect to Q if and only if at least μ*|*S*| *vertices of S are contained in Q*.

Henceforth, *μ*-enriched *γ*-quasi-cliques will hereafter be referred to as *μ*, *γ*-quasi-cliques, and the "query" set of vertices will be denoted as *Q*.

**Definition 1.3 ***Given a labeled graph G, a "query" set of vertices Q, a real value γ *??#8712; (0.5, 1], *and a real value μ *??#8712; (0, 1], *a γ-dense quasi-clique S is also **maximal **if no larger supergraph S' of S is a γ-dense quasi clique that is μ-enriched with respect to Q*.

The algorithm to enumerate *μ*, *γ*-quasi-cliques is an agglomerative bottom-up approach with a backtracking paradigm. The basic premise of the algorithm is that we will build the *μ*, *γ*-quasi-cliques starting with a single query vertex *v*_0 _(*v*_0 _??#8712; *Q*) and backtracking as we find maximal *μ*, *γ*-quasi-cliques or subgraphs that cannot be contained in a *μ*, *γ*-quasi-clique. For this section, we use the convention that *S *represents the current subgraph under consideration, and *C *represents the set of vertices that could extend *S *to produce a *μ*, *γ*-quasi-clique. The number of vertices in *S *adjacent to a vertex *v *is denoted as *s_a_*(*v*) and in *C *is denoted as *c_a_*(*v*). *N^k^*(*S*) denotes all vertices at distance *k *(*k *edges) or less from all vertices of *S*. To improve the efficiency of the algorithm we use some theoretical results and properties (the detailed proofs are available in Supplement 1). The properties are targeted at three points to improve efficiency (1) reducing the size of *C*, i.e., the search space of candidates be added, (2) deciding on when to stop expanding a subgraph *S *further, and (3) deciding on when to discard a subgraph *S *if it can never be a *μ*, *γ*-quasi-clique. The first property is based on a result presented by Pei *et al *[37], it states that for *S *to be a *μ*, *γ*-quasi-clique, every pair of vertices has to be at a maximum distance of 2 edges from each other. Using this property, the size of the candidate set *C *for any subgraph *S *can at the maximum only have |*N*^2^(*S*)|*/*|*S*| entries. The second property based on results drawn from Zeng *et al *[38] states that if for any given vertex *v *??#8712; *V *(*S*), the number of vertices in *C *and *S *that are adjacent to *v *together do not satisfy the *γ *constraint, then no supergraph of *S *will ever satisfy the *γ *constraint, i.e., *s_a_*(*v*) + *c_a_*(*v*) *> γ*(|S| - 1 + *c_a_*(*v*)) needs to be satisfied to warrant expanding *S *further; otherwise, we output *S *as the maximal *μ*, *γ*-quasi-clique. The third property states that for any vertex *v *??#8712; *C*, *S *??#8746; {*v*} or any supergraph of *S *??#8746; {*v*} can satisfy the *γ *criterion if and only if *s_a_*(*v*) + *c_a_*(*v*) ≥ *γ *(|*S*| + *c_a_*(*v*)). All vertices in *C *that do not satisfy this constraint can be removed from the candidate list, thereby reducing the search space further. The fourth property deals with reducing the size of *C *based on the enrichment constraint. The current subgraph *S *is *μ*-enriched if |*S *??#8745; *Q*| ≥ *μ*|*S*|. The condition |*S *??#8745; *Q*| + |*C *??#8745; *Q*| ≥ *μ*(|*S*| + |*C *??#8745; *Q*|) must be met by every *S *that can be further extended and still satisfy the *μ *criterion. The maximum increase in enrichment occurs when subgraph *S *is extended by the addition of all vertices from *C *??#8745; *Q*. This maximum enrichment has to be less than the sum of the number of vertices common between *Q *and *S*, and *Q *and *C*, to warrant any further expansion of *S*. If during the algorithm execution we reach a point where the addition of a vertex *v *to the current subgraph *S*' results in a subgraph *S *that violates the above condition, *v *is removed from the candidate list. Additional properties for restricting the search space of potential *μ*, *γ*-quasi-cliques are available in Supplement 1. We loop through all vertices in the query set *Q *and for each vertex *v *??#8712; *Q *we enumerate all the *μ*, *γ*-quasi maximal cliques that contain *v *and avoid enumerating the same subgraph twice by keeping track of the ones enumerated earlier. All the above theoretical properties and results are utilized to improve the efficiency of the backtracking algorithm (The detailed pseudocode is available Additional File 3). In order to decide when a *μ*, *γ*-quasi-clique is maximal, we propose to maintain a bitmap index of the *μ*, *γ*-quasi-cliques that contains each vertex. As the algorithm identifies *μ*, *γ*-quasi-cliques, it assigns numbers to them sequentially and adds these values to indices for the vertices contained in the *μ*, *γ*-quasi-cliques. Then, as we add and remove vertices from set *C*, we check these bitmap indices to see if there is an already-discovered *μ*, *γ*-quasi-clique that contains all vertices of *S *??#8746; *C *by performing a bitwise and of the indices associated with the vertices of *S *??#8746; *C*. If there is an already-discovered *μ*, *γ*-quasi-clique that is a superset of *S *??#8746; *C*, we may safely backtrack, as no further extensions of *S *will be maximal. One drawback of using a bitmap index, however, is that as more *μ*, *γ*-quasi-cliques are identified, the size of the index will increase. In an effort to avoid checking the entire index for each vertex (in the case where *S *??#8746; *C *is maximal), we propose using a hierarchical bitmap index, in which each byte of the index is summarized by a single bit in a higher level index. As we are checking for the existence of a bit that is set in all of the indices related to the vertices of *S *??#8746; *C*, we do not need to examine bytes that have no bits set. As such, we summarize zero bytes in the "base level" index with a 0 and nonzero bytes with a 1. As the size of the index grows, we can add more levels, summarizing each byte in the "first level" index with a bit in the "second level" index, each byte in the "second level" index with a bit in the third, and so on. In this way, we can use higher level indices to reduce the number of bytes we need to check on the "base level" index.

### Parameter Selection

DENSE requires the user input of two parametes: the enrichment (*μ*) and the density (*γ*). The earlier description of these parameters suggests that higher values of *γ *will produce more connected (clique-like) subgraphs. Similarly, higher values of the enrichment (*μ *≥ 0.5) will produce subgraphs that are primarily composed of the "query" vertices, whereas a very low value (*μ *≤ 0.001) will result in enumeration of all the subgraphs that satisfy the *γ *threshold and contain at least one query vertex.

Parameter thresholds depend on the application. In this paper, we are interested in identifying phenotype-related protein functional modules, given a user-defined initial set of phenotype-related proteins as a query. Setting *μ *value to 0.001 will result in finding all the modules that could potentially be related to phenotype-expression (e.g., via guilt-by-association). Since a functional module is believed to form a group of highly connected proteins in a protein functional association network [43], the authors of [44,45] suggested that the density of the subgraph that represents a functional module should fall between 0.5 and 1, where the greater the density is, the more likely the subgraph is a true functional module. Based on these observations, setting *γ *= 1 will produce those subgraphs that are the most probable functional modules. However, since organismal networks are prone to missing information (edges), the value of *γ *= 1 could be too stringent, and the algorithm may miss some of the phenotype-related modules. Hence, we chose a *γ *value of 0.75 (midpoint of 0.5 and 1) to identify highly connected (but not fully connected) subgraphs as most probable modules that are functionally associated with phenotype-related query proteins.

## Authors' contributions

WH developed the underlying theory and the computational model and implemented the algorithm. WH and KP conducted computational experiments. AR and KS provided biological validation. WH, KP, and AR provided the initial draft of the manuscript. JM suggessted and supervised the study related to the hydrogen production from wastewater and waste materials. NFS provided the problem statement, supervised the developement of the computational methodology, and provided suggesstions on methodology validation. JM, KS, AC, and NFS contributed to preparing the final version of the manuscript. All authors have read and approved the final manuscript.

## Supplementary Material

Additional file 1 **Dark Fermentation Phenotype Results**. The file contains the results of the dark fermentation, hydrogen production experiment. Click here for file

Additional file 2 **Acid-tolerance Phenotype Results**. The file contains the results of the acid-tolerance experiment. Click here for file

Additional file 3 **Additional Method Details**. This file contains the proofs of the various properties and results used in the method section. It also has the detailed pseudocode for the algorithm along with some description on where in the pseudocode the theoretical results are used. Click here for file
