# Supplementary material for: DENSE: efficient and prior knowledge-driven discovery of phenotype-associated protein functional modules
Source: BMC Syst Biol. 2011 Oct 24;5:172. doi: 10.1186/1752-0509-5-172 (PMC3231954; doi:10.1186/1752-0509-5-172)
Supplement: Additional file 3 — Additional Method Details. This file contains the proofs of the various properties and results used in the method section. It also has the detailed pseudocode for the algorithm along with some description on where in the pseudocode the theoretical results are used. [file 1752-0509-5-172-S3.PDF]

The various theorotical results stated in the paper are discussed here in detail with proofs where necessary. Our first result, originally presented in [1] as a special case of Theorem 1, places a limit on the *diameter*, or the maximum length of the shortest path between two vertices, of a μ, γ-quasi-clique. As this result has already been presented, the proof is omitted here.

Theorem 0.1 *Let S be a γ-dense quasi-clique where $\gamma \geq 0.5$. The diameter of S is at most 2.*

Corollary 0.1 *Let S be a subgraph of G. Any γ-dense quasi-clique that is a supergraph of S will consist of vertices at most distance 2 from every vertex of S. We denote this set as $N^2(S)$.*

For the rest of the results presented in this section, we will adopt the convention that S represents the current subgraph and C represents the set of "candidate" vertices that could be added to S to form a μ, γ-quasi-clique. By the result of Corollary 0.1, we can see that the set $N^2(S) \setminus S$ contains every vertex that could be added to S to form a μ, γ-quasi-clique, and as such, $C = N^2(S) \setminus S$ represents a good starting point for the candidate set.

Several of the following theorems are designed to reduce the size of C, eliminating unfruitful branches of the search tree as soon as possible. Theorems 0.2 and 0.3 use the γ density requirement to place bounds on how "connected" a vertex must be to be contained in a μ, γ-quasi-clique. Intuitively, the greatest possible percentage of vertices in S adjacent to a vertex v would occur if we moved to S the vertices adjacent to v in C, and if the resulting subgraph doesn't satisfy the γ density requirement on v, no supergraph of S will do so. These results are drawn from Lemmas 5.4–5.6 in [2], but a proof is presented here for clarity.

Theorem 0.2 *Let S be a subgraph of G, and let C be the set of all possible vertices that may be contained in some supergraph of S that is a γ-dense quasi-clique. Let v be any vertex of S, and let s_a and c_a be the number of vertices of S and C, respectively, that are adjacent to v. If $s_a + c_a < \gamma(|S| - 1 + c_a)$, no supergraph of S can be a γ-dense quasi-clique.*

Proof 0.1 *Suppose the negation: $s_a + c_a < \gamma(|S| - 1 + c_a)$ and there exists a supergraph H of S that is a γ-dense quasi-clique.*

Let h_a be the number of vertices of $H \setminus S$ adjacent to v. By the definition of the set C, $h_a \leq c_a$. Subtracting $\gamma(c_a - h_a)$ from both sides of the previous inequality, we see that $s_a + c_a - \gamma(c_a - h_a) < \gamma(|S| - 1 + h_a)$. As $\gamma > 0$ and $c_a - h_a \geq 0$, $c_a - h_a \geq \gamma(c_a - h_a)$, so

$$\begin{aligned} s_a + h_a &= s_a + c_a - (c_a - h_a) \\ &\leq s_a + c_a - \gamma(c_a - h_a) \\ &< \gamma(|S| - 1 + h_a) \end{aligned}$$

Since H must have at least $|S| + h_a$ vertices, $\gamma(|S| - 1 + h_a) \leq \gamma(|H| - 1)$, so $s_a + h_a < \gamma(|H| - 1)$. However, H has only $s_a + h_a$ vertices adjacent to v, implying that H is not a γ-dense quasi-clique. This is a contradiction; therefore, the claim must be true.

Theorem 0.3 *Let S be a subgraph of G, and let v be a vertex in C. Let s_a be the number of vertices of S adjacent to v, and let c_a be the number of vertices of C adjacent to v. If $s_a + c_a \leq \gamma(|S| + c_a)$, neither $S \cup \{v\}$ nor any supergraph of it can be a γ-dense quasi-clique.*

Proof 0.2 *The proof is similar to the proof for Theorem 0.2.*

Much as the previous two results establish bounds based on the density requirement for μ, γ-quasi-cliques, Theorem 0.4 allows us to prune the set of "candidate" vertices based on the enrichment requirement. The intuition behind the theorem is that S would not satisfy the μ enrichment criterion after moving all the vertices in C that are also in Q to S, no supergraph of S will be able to satisfy the μ enrichment criterion.

Theorem 0.4 *Let c_q be the number of vertices in $C \cap Q$. If there are fewer than $\mu|S| - (1 - \mu)c_q$ vertices in $S \cap Q$, then neither S nor any supergraph will be μ-enriched.*

Proof 0.3 *Suppose not, and let H be a supergraph of S that is a μ-enriched. Let h_q be the number of vertices of $H \setminus S$ that are in Q. Since C contains c_q vertices in Q, $h_q \leq c_q$. As S contains less than $\mu|S| - (1 - \mu)c_q$ vertices in Q, H contains less than $\mu|S| - (1 - \mu)c_q + h_q \leq \mu|S| - (1 - \mu)h_q + h_q = \mu(|S| + h_q)$ vertices in Q. However, as $|H| \geq |S| + h_q$, this implies that H contains less than $\mu|H|$ vertices in Q, contradicting our assumption that H is μ-enriched.*

Finally, Theorems 0.5–0.8 establish restrictions on building μ, γ-quasi-cliques through a combination of the density and enrichment requirements. The intuitions here are that after adding all of the candidate vertices that are in Q and are adjacent to a vertex, we are limited in the number of other (non-query) adjacent vertices that we can add by the enrichment condition, and the density condition limits the number of other (non-adjacent) query vertices we can add (Theorems 0.5 and 0.6).

Lemma 0.1 *Let H be a μ, γ-quasi-clique, let S be a subgraph of H, and let v be a vertex of S. Let s_a be the number of vertices of S adjacent to v, $s_{\overline{q}}$ be the number of vertices in S that are not in Q, c_{aq} be the number of vertices in $H \setminus S$ that are in Q and adjacent to v, and $c_{a\overline{q}}$ be the number of vertices in $H \setminus S$ that are adjacent to v but not in Q.*

$$(1 - \mu)(s_a + c_{aq} + c_{a\overline{q}} + \gamma) - \gamma(s_{\overline{q}} + c_{a\overline{q}}) \geq 0$$

Proof 0.4 *H must contain at least $\mu|H|$ vertices in Q, so it contains at most $|H| - \mu|H| = (1-\mu)|H|$ vertices not in Q. By our definitions, $s_{\overline{q}}$ and $c_{a\overline{q}}$ both represent vertices in H that are not in Q, so this fact implies that*

$$s_{\overline{q}} + c_{a\overline{q}} \leq (1-\mu)|H|. \tag{1}$$

Similarly, H must contain at least $\gamma(|H|-1)$ vertices adjacent to v, so

$$\gamma(|H|-1) \leq s_a + c_{aq} + c_{a\overline{q}}, \text{ or} \tag{2}$$
$$\gamma|H| \leq s_a + c_{aq} + c_{a\overline{q}} + \gamma. \tag{3}$$

Multiplying equation 1 by γ and equation 3 by $1-\mu$ (both of which must be positive), we see that

$$\gamma(s_{\overline{q}} + c_{a\overline{q}}) \leq \gamma(1-\mu)|H| \text{ and} \tag{4}$$
$$\gamma(1-\mu)|H| \leq (1-\mu)(s_a + c_{aq} + c_{a\overline{q}} + \gamma). \tag{5}$$

Thus, $\gamma(s_{\overline{q}} + c_{a\overline{q}}) \leq (1-\mu)(s_a + c_{aq} + c_{a\overline{q}} + \gamma)$, proving the claim.

Theorem 0.5 *Let v be a vertex in S, and let s_a, $s_{\overline{q}}$, c_{aq}, and $c_{a\overline{q}}$ be as in Lemma 0.1, except with c_{aq} and $c_{a\overline{q}}$ being vertices in C rather than $H \setminus S$. If $\gamma < 1-\mu$ and $(1-\mu)(s_a + c_{aq} + c_{a\overline{q}} + \gamma) - \gamma(s_{\overline{q}} + c_{a\overline{q}}) < 0$, then neither S nor any supergraph of S will be a μ, γ-quasi-clique.*

Proof 0.5 *Consider an arbitrary μ, γ-quasi-clique H such that S is a subgraph of H. If we let h_{aq} and $h_{a\overline{q}}$ represent the number of vertices in $H \setminus S$ that are adjacent to v and are in Q and not in Q, respectively, then h_{aq} and $h_{a\overline{q}}$ must satisfy the inequality $(1-\mu)(s_a + h_{aq} + h_{a\overline{q}} + \gamma) - \gamma(s_{\overline{q}} + h_{a\overline{q}}) \geq 0$ by Lemma 0.1. As H must be a subgraph of $S \cup C$, h_{aq} and $h_{a\overline{q}}$ must satisfy $0 \leq h_{aq} \leq c_{aq}$ and $0 \leq h_{a\overline{q}} \leq c_{a\overline{q}}$. As $\gamma < 1-\mu$, $(1-\mu)(s_a + h_{aq} + h_{a\overline{q}} + \gamma) - \gamma(s_{\overline{q}} + h_{a\overline{q}})$ is maximized at $h_{aq} = c_{aq}$ and $h_{a\overline{q}} = c_{a\overline{q}}$, so if $(1-\mu)(s_a + c_{aq} + c_{a\overline{q}} + \gamma) - \gamma(s_{\overline{q}} + c_{a\overline{q}}) < 0$, no subgraph of $S \cup C$ containing v may be a μ, γ-quasi-clique.*

Theorem 0.6 *Let v be a vertex in S, and let s_a, $s_{\overline{q}}$, and c_{aq} be as in Lemma 0.1, except with c_{aq} being vertices in C rather than $H \setminus S$. If $\gamma \geq 1-\mu$ and $(1-\mu)(s_a + c_{aq} + \gamma) - \gamma s_{\overline{q}} < 0$, then neither S nor any supergraph of S will be a μ, γ-quasi-clique.*

Proof 0.6 *The proof for Theorem 0.6 is similar to the proof for Theorem 0.5, with the exception that $(1-\mu)(s_a + h_{aq} + h_{a\overline{q}} + \gamma) - \gamma(s_{\overline{q}} + h_{a\overline{q}})$ is maximized at $h_{aq} = c_{aq}$ and $h_{a\overline{q}} = 0$.*

Lemma 0.2 *Let H be a μ, γ-quasi-clique, let S be a subgraph of H, and let v be a vertex of S. Let s_q be the number of vertices of S in Q, $s_{\overline{a}}$ be the number of vertices in $S \setminus \{v\}$ not adjacent to v, c_{aq} be the number of vertices in $H \setminus S$ that are adjacent to v and in Q, and $c_{\overline{a}q}$ be the number of vertices in $H \setminus S$ that are in Q but not adjacent to v.*

$$(1-\gamma)(s_q + c_{aq} + c_{\overline{a}q}) - \mu(s_{\overline{a}} + c_{\overline{a}q} + 1 - \gamma) \geq 0$$

Proof 0.7 *This lemma follows from the inequalities* $(1-\gamma)(|H|-1) \leq s_{\overline{a}} + c_{\overline{a}q}$ *and* $\mu|H| \leq s_q + c_{aq} + c_{\overline{a}q}$*, similar to Lemma 0.1.*

Theorem 0.7 *If* $\gamma < 1-\mu$ *and* $(1-\gamma)(s_q + c_{aq} + c_{\overline{a}q}) - \mu(s_{\overline{a}} + c_{\overline{a}q} + 1 - \gamma) < 0$*, then neither* S *nor any supergraph of* S *will be a* μ, γ*-quasi-clique.*

Theorem 0.8 *If* $\gamma \geq 1-\mu$ *and* $(1-\gamma)(s_q + c_{aq}) - \mu(s_{\overline{a}} + 1 - \gamma) < 0$*, then neither* S *nor any supergraph of* S *will be a* μ, γ*-quasi-clique.*

Proof 0.8 *Similar to Theorems 0.5 and 0.6, Theorems 0.7 and 0.8 follow from maximizing the expression* $(1-\gamma)(s_q + h_{aq} + h_{\overline{a}q}) - \mu(s_{\overline{a}} + c_{\overline{a}q} + 1 - \gamma)$ *at* $h_{aq} = c_{aq}$ *and* $h_{\overline{a}q} = c_{\overline{a}q}$ *or 0, respectively. (Note that* $\mu < 1-\gamma$ *iff* $\gamma < 1-\mu$*.)*

1 Algorithm

Algorithm 1: Pseudocode outline of the μ, γ-quasi-clique algorithm

foreach $v_0 \in Q$ **do**
    $S \leftarrow \{v_0\}$;
    $C \leftarrow N^2(v_0)$;
    Calculate e;
    Calculate d_v for all $v \in S \cup C$;
    Calculate g_v for all $v \in S \cup C$;
    Calculate m_v for all $v \in S \cup C$;
    Remove all unpromising vertices of C;
    if $S \cup C$ *is maximal* **then**
        Enumerate;
end

From Corollary 0.1, we can see that $N^2(S)$ can serve as an appropriate starting point for our set C. However, rather than recalculate this intersection of sets every time a vertex is added to the set S, we first define C as the set of all vertices within distance 2 of the inital vertex, $N^2(v_0)$, and intersect C with $N^2(v)$ for each vertex v we add to S. As these $N^2(v_0)$ sets can be precomputed and stored in a matrix, this should make for a much more efficient way to apply Corollary 0.1.

By Theorem 0.3, we know that for any vertex $v \in C$, if s_a represents the number of vertices of S adjacent to v, c_a represents the number of vertices of C adjacent to v, and $s_a + c_a \leq \gamma(|S| + c_a)$, then neither $S \cup \{v\}$ nor any supergraph can be a μ, γ-quasi-clique. Rather than recomputing this inequality every time we add or remove a vertex from S, we calculate and maintain the value of $s_a + c_a - \gamma(|S| + c_a)$ as d_v—when this value becomes zero or negative, v may be removed from C. Thus, at the outset of the algorithm, we calculate the values of $s_a + (1-\gamma)c_a$ for each $v \in C$. When a vertex $u \in C$ is added to S, d_v will remain unchanged if u is adjacent to v, but d_v will decrease by γ if u

Algorithm 2: Pseudocode for `Enumerate` function

```
$T \leftarrow C$;
while some vertices of $C$ are marked do
    Remove all marked vertices from $C$;
    if $S$ violates one of the theoretical constraints then
        Restore all vertices of $T \setminus C$ to $C$;
        return;
    end
    Update $e$ and all $d_v$, $g_v$, and $m_v$ values as appropriate;
    if $S \cup C$ is nonmaximal then
        Backtrack until some vertex of $C$ is restored;
end
while $C \neq \emptyset$ do
    Choose $v$ in $C$ according to some heuristic and move $v$ to $S$;
    Update $e$ and all $d_v$, $g_v$, and $m_v$ values as appropriate;
    if $g_v < 0$ or $m_v < 0$ for some $v \in S$ then
        Restore vertices of $T \setminus C$ to $C$;
        Update $e$, $d_v$, $g_v$, and $m_v$ values appropriately;
        return;
    end
    Mark all vertices of $C$ to be removed;
    if $S$ does not violate any of the theoretical constraints then
        Enumerate;
    Remove $v$ from $S$;
    Update $e$ and all $d_v$, $g_v$, and $m_v$ values as appropriate;
    if $S$ violates one of the theoretical constraints then
        Restore vertices of $T \setminus C$ to $C$;
        Update $e$ and all $d_v$, $g_v$, and $m_v$ values;
        return;
    end
    Iteratively remove unpromising vertices of $C$;
    Update $e$ and all $d_v$, $g_v$, and $m_v$ values as appropriate;
    if $S \cup C$ is nonmaximal then
        Backtrack until some vertex of $C$ is restored;
end
if no recursive call of Enumerate() found a $\mu, \gamma$-quasi-clique then
    Output $S$;
    Update the maximality index for each vertex in $S$;
end
Restore vertices of $T \setminus C$ to $C$;
Update $e$, $d_v$, $g_v$, and $m_v$ values appropriately;
return;
```

Table 1: Values by which d_v is decreased when a vertex $u \in C$ is moved to S or removed from C

	$u \in N(v)$	$u \notin \overline{N(v)} \cup \{v\}$	$u = v$
u moved to S	0	γ	0
u removed from C	$1-\gamma$	0	–

Table 2: Values by which e is decreased when a vertex $u \in C$ is moved to S or removed from C

	$u \in Q$	$u \notin Q$
u moved to S	0	μ
u removed from C	$1-\mu$	0

is not adjacent to v. Similarly, d_v increases by γ when a vertex nonadjacent to v is removed from S. When a vertex $u \in C$ is removed from C, d_v will decrease by $1-\gamma$ if u is adjacent to v, but it will be unaffected if u is nonadjacent to v. Similarly, d_v will increase by $1-\gamma$ when u is returned to C.

By Theorem 0.2, we know that for vertices $v \in S$, $s_a + c_a \leq \gamma(|S| - 1 + c_a)$, where s_a and c_a are defined as before. As such, we maintain a d_v value for each $v \in S$ just as we maintain d_v values for vertices in C (see Table 1). Finally, to calculate d_v when v is moved from C to S, we use the same value of d_v as when v was in the set C. (The value of d_v is unimportant if v is removed from C.) Table 1 summarizes the values by which d_v is updated.

When the value of d_u becomes negative for a vertex $u \in C$, we can remove u from C by the result of Theorem 0.3. Additionally, when d_v decreases below γ for a vertex $v \in S$, we can remove all vertices of C that are nonadjacent to v, as adding such vertices to S would violate Theorem 0.2.

In a similar fashion, we calculate the initial values for e and each g_v and m_v value and update these values as the algorithm progresses. We can then remove vertices from C whose addition to C would violate Theorems 0.4, 0.5, 0.6, 0.7, or 0.8. Tables 2, 3, and 4 summarize the amount by which the e, g_v, and m_v values are updated as vertices are moved from C to S or removed from C.

Table 3: Table summarizing values by which g_v decreases when a vertex $u \in C$ is moved to S (column "M") or removed from C (column "R")

u	$N(v), Q$		$N(v), \neg Q$		$\neg N(v), Q$		$\neg N(v), \neg Q$	
	M	R	M	R	M	R	M	R
$\gamma \geq 1-\mu$	0	$1-\mu$	$\gamma-(1-\mu)$	0	0	0	γ	0
$\gamma < 1-\mu$	0	$1-\mu$	0	$1-\mu-\gamma$	0	0	γ	0

Table 4: Table summarizing values by which m_v decreases when a vertex $u \in C$ is moved to S (column "M") or removed from C (column "R")

u	$N(v), Q$		$N(v), \neg Q$		$\neg N(v), Q$		$\neg N(v), \neg Q$	
	M	R	M	R	M	R	M	R
$\gamma \geq 1-\mu$	0	$1-\gamma$	0	0	$\mu-(1-\gamma)$	0	μ	0
$\gamma < 1-\mu$	0	$1-\gamma$	0	0	0	$1-\gamma-\mu$	μ	0

References

[1] J. Pei, D. Jiang, and A. Zhang. Mining cross-graph quasi-cliques in gene expression and protein interaction data. In *Proceedings, 21st International Conference on Data Engineering (ICDE 2005)*, pages 353–356, April 2005.

[2] Zhiping Zeng, Jianyong Wang, Lizhu Zhou, and George Karypis. Out-of-core coherent closed quasi-clique mining from large dense graph databases. *ACM Trans. Database Syst.*, 32(2):13, 2007.
